# *Polygonatum kingianum* polysaccharide alleviated intestinal injuries by mediating antioxidant ability and microbiota

**DOI:** 10.3389/fmicb.2025.1492710

**Published:** 2025-01-30

**Authors:** Reng Qiu, Chuangye Pan, Yuxi Qin, Qianfei Wei, Yue Yu, Ying Zhang, Xuehan Xie, Jianqin Li, Shouhai Chen, Kun Li, Dalia Fouad, Yi Wu, Qiu Zhong

**Affiliations:** ^1^Henan Provincial Engineering Laboratory of Insects Bio-reactor, Henan Provincial Engineering and Technology Center of Health Products for Livestock and Poultry, Nanyang Normal University, Nanyang, Henan, China; ^2^Guangxi Key Laboratory of Animal Breeding, Disease Control and Prevention, College of Animal Science and Technology, Guangxi University, Nanning, Guangxi, China; ^3^College of Veterinary Medicine, Yunnan Agricultural University, Kunming, Yunnan, China; ^4^College of Veterinary Medicine, Nanjing Agricultural University, Nanjing, Jiangsu, China; ^5^Department of Zoology, College of Science, King Saud University, Riyadh, Saudi Arabia

**Keywords:** *Escherichia*, *Pseudomonas*_E, *Mailhella*, *Paramuribaculum*, NM07-P-09, *Odoribacter*, *Nanosyncoccus*, SFMI01

## Abstract

**Introduction:**

*Polygonatum kingianum* is a well-known medicinal herb with proven bioactivities; however, little is known about the effects of its polysaccharide on intestinal injuries in animals induced by lipopolysaccharide (LPS).

**Methods:**

A total of 30 Institute of Cancer Research (ICR) mice were divided into control (CH), induced (MH), and treated (H) groups. Mice in group H were supplemented with 100 mg/kg *Polygonatum kingianum* polysaccharides, while groups C and M were treated with the same amount of normal saline by gavage for 18 days. On the 18^th^ day animals in groups M and H were induced by LPS (10 mg/kg).

**Results:**

The results showed the weight of mice in group MH significantly dropped (*P* < 0.0001), while mice in the PK group had a higher weight (*P* < 0.01). Pathological analysis found that the majority of the villi in mice induced by LPS were broken and short, while PK-treated animals had longer and considerably integrated villi. The villi length in groups CH (*P* < 0.0001) and H (*P* < 0.0001) was longer than that in group M, and the value of villi length/crypt depth in group MH was smaller than that in groups CH (*P* < 0.0001) and H (*P* < 0.0001), while the crypt depth in group MH was higher than in groups CH (*P* < 0.0001) and H (*P* < 0.0001). Serum inspection showed that MAD (*P* < 0.05), IL-1β (*P* < 0.05), IL-6 (*P* < 0.05), and TNF-α (*P* < 0.01) were significantly higher in group MH, while SOD (*P* < 0.001), T-AOC (*P* < 0.01), and GSH-Px (*P* < 0.01) were notably higher in groups CH and H. Microbiome sequencing of mice obtained 844,477 raw and 725,469 filtered reads. There were 2,407 ASVs detected in animals, and there were 312 and 328 shared ASVs between CH and MH, and CH and H, respectively. There were 5 phyla and 20genera of remarkable bacteria found among mice groups including genera of *Escherichia, Pseudomonas_E, Mailhella, Paramuribaculum*, NM07-P-09, *Odoribacter, Nanosyncoccus*, SFM01, *Onthenecus, Clostridium_Q*, UBA6985, *Ructibacterium*, UBA946, *Lachnoclostridium_B, Evtepia*, CAG-269, *Limivicinus, Formimonas, Dehalobacterium, Dwaynesavagella*, and UBA6985. We revealed that Polygonatum kingianum polysaccharide could alleviate intestinal injuries by promoting oxidation resistance, decreasing inflammatory responses, and accommodating the intestinal microbiota of mice.

**Discussion:**

Our results suggest the possibility of developing novel therapies for intestinal diseases.

## 1 Introduction

The intestine is a complex organ that plays a crucial role in digestion, nutrient assimilation, immune function, and overall host health (Hickey et al., 2023). Intestinal injury is a common symptom of many conditions, including radiation therapy-related diseases (Lin et al., 2023), heat stress (Li et al., 2020), inflammatory bowel disease (Li H. et al., 2023; Li et al., 2023a,b), and infectious diseases (Donaldson et al., 2022). However, treating intestinal damage remains challenging, and novel therapies are urgently needed (Ling et al., 2024; Cho et al., 2021; Yang et al., 2022).

Traditional Chinese medicine (TCM) has been used effectively to treat different diseases in history (Huang et al., 2021), such as ulcerative colitis (Liu et al., 2022), liver disease (Zhu et al., 2023), and inflammatory bowel disease (Yang L. et al., 2021). *Polygonatum kingianum* is a well-known medicinal herb in TCM, grown in Yunnan, China. It is known for its bioactivities, including immuno-enhancement, anti-aging, anti-fatigue properties, and the regulation of glucolipid metabolism (Dong et al., 2021; Li X. et al., 2023). There are several effective constituents in this herb-like plant: polysaccharides, saponins, phenolics, and flavonoids (Yang et al., 2020). Previous studies found that *Polygonatum kingianum* polysaccharide (PKP) could promote glucolipid metabolism (Gu et al., 2020), regulate microbiota and short-chain fatty acids (Yang M. et al., 2021), and enhance immunity (Su et al., 2023).

The gut microbiota is composed of billions of microorganisms including prokaryotes and eukaryotes (Lozupone et al., 2012), which play important roles in the host's immunity, nutrient absorption, metabolism, and overall health (Adak and Khan, 2019). Gut dysbiosis was associated with inflammatory, metabolic, and neurodegenerative diseases (Levy et al., 2017), Crohn's disease (Caparrós et al., 2021), and inflammatory bowel diseases (Haneishi et al., 2023). Lipopolysaccharide is a crucial adjective membrane structural component of Gram-negative microbes (Sweeney and Lowary, 2019), which can stimulate immune response and promote the secretion of pro-inflammatory factors (Mohr et al., 2022). Previous studies reported that LPS caused intestinal damage and intestinal flora disorder (Izadparast et al., 2022; Yan et al., 2022). However, little information is available regarding the influence of *Polygonatum kingianum* polysaccharide on intestinal injuries in animals induced by LPS. Therefore, this study was conducted to investigate the mediating effect of PKP on mice challenged by LPS by regulating antioxidant ability and microbiota.

## 2 Materials and methods

### 2.1 Animal experiment

Thirty ICR mice (4 weeks, 22.81 ± 0.98 g) with the same amount of male and female animals were procured from the experimental animal center at Yangzhou University. All those rodents were given 3 days for acclimatization and then divided into control (CH), induced (MH), and treated (H) groups. Mice in the H group were supplemented with 100 mg/kg *Polygonatum kingianum* polysaccharide (Yuanye Bio-Technology Co., Ltd, Shanghai, China), while the C and M groups were treated with an even volume of normal saline by gavage for 18 days. On the 18th, animals in the M and H groups were induced by LPS (10 mg/kg) (Meng et al., 2024; Peng et al., 2024), and then those animals were euthanized to obtain blood and intestine samples the next day.

### 2.2 Pathological staining

Approximately 1–2 cm tissue samples of the jejunum and ileum from mice in the CH, MH, and H groups were kept in formaldehyde solution (4%) for more than 2 days and then used for H&E staining in Wuhan Pinuofei Biological Technology (China). Pathological examination was performed using Olympus CX23 (Olympus Co., Japan), and the villus height and crypt depth of enteric samples were measured as per the guidelines of published studies (He et al., 2023; Chen et al., 2022).

### 2.3 Serum oxidation resistance and inflammation levels detection

All the mice blood samples were centrifuged at 4,000 *g* x 8 min to isolate serums for oxidation resistance and inflammation level detection using assay kits of malonaldehyde; superoxide dismutase; total anti-oxidation capacity; glutathione peroxidase; interleukins-−1β, IL-6, and IL-10; and tumor necrosis factor alpha bought from Jiancheng Bioengineering Research Institute (Nanjing, China) and Solarbio Life Science (China).

### 2.4 Flora sequencing

The microbial DNA of rectal contents from three mice in each group was extracted by applying a stool genomic DNA extraction kit (Solarbio, China). The product quality was then confirmed using a NanoDrop One^C^ (Thermo Scientific, USA) and agarose gel electrophoresis (1.2%), as previously described in studies by Mattei et al. (2019) and Zhang X. et al. (2022). The V3-4 gene of 16S rRNA of the microbes in mice in different groups was magnified by piloting objective primers 338F/806R (F: 5′-ACTCCTACGGGAGGCAGCAG-3′, R:5′-GGACTACHVGGGTWTCTAAT-3′) (Yu et al., 2020). Finally, sequencing libraries for mice from the C, M, and H groups were constructed using an NGS Tn5 DNA library prep kit (Ruikang Technology Co., LTD, Beijing, China) for further sequencing through the Illumina platform at Bioyi Biotechnology Co., Ltd. (Wuhan, China) (Kong et al., 2024).

### 2.5 Sequencing data analysis

The raw sequencing data from mice in groups C, M, and H were quality-filtered using DADA2 (Callahan et al., 2016), and characteristic tabulation of amplicon sequence variant (ASV) was generated using QIIME2 (Nagarajan et al., 2023). The co-existing ASVs were examined and presented using a Venn map (Dou et al., 2023). ANOVA and LEfSe were used to screen markedly different bacterial taxa between rodent animal groups (Wells et al., 2022; Lv et al., 2023). The diversity analysis of alpha (observed species, Chao1, Shannon, Simpson, Faith's PD, and Pielou's evenness) and beta diversity (principal coordinate analysis and non-metric multidimensional scaling) analyses were performed to estimate the bacterial diversity, evenness, and richness of samples and group structure variation in mice samples using QIIME2 (Long et al., 2023; Zhong et al., 2023; Deng et al., 2021; Zhang Y. et al., 2022). Finally, the microflora function was predicted using PICRUSt2 (Li et al., 2023a,b), and functional differences among the CH, MH, and H groups were examined by annotating with the KEGG and MetaCyc databases.

### 2.6 Statistical analysis

Variances among the CH, MH, and H groups were assessed using ANOVA and Student's *t*-test relying on IBM SPSS (26.0). The mice data were presented as means ± standard deviation (SD), with *P* < 0.05 considered statistically significant.

## 3 Results

### 3.1 PK relieved intestinal damage in mice caused by LPS

**Figure 1:**
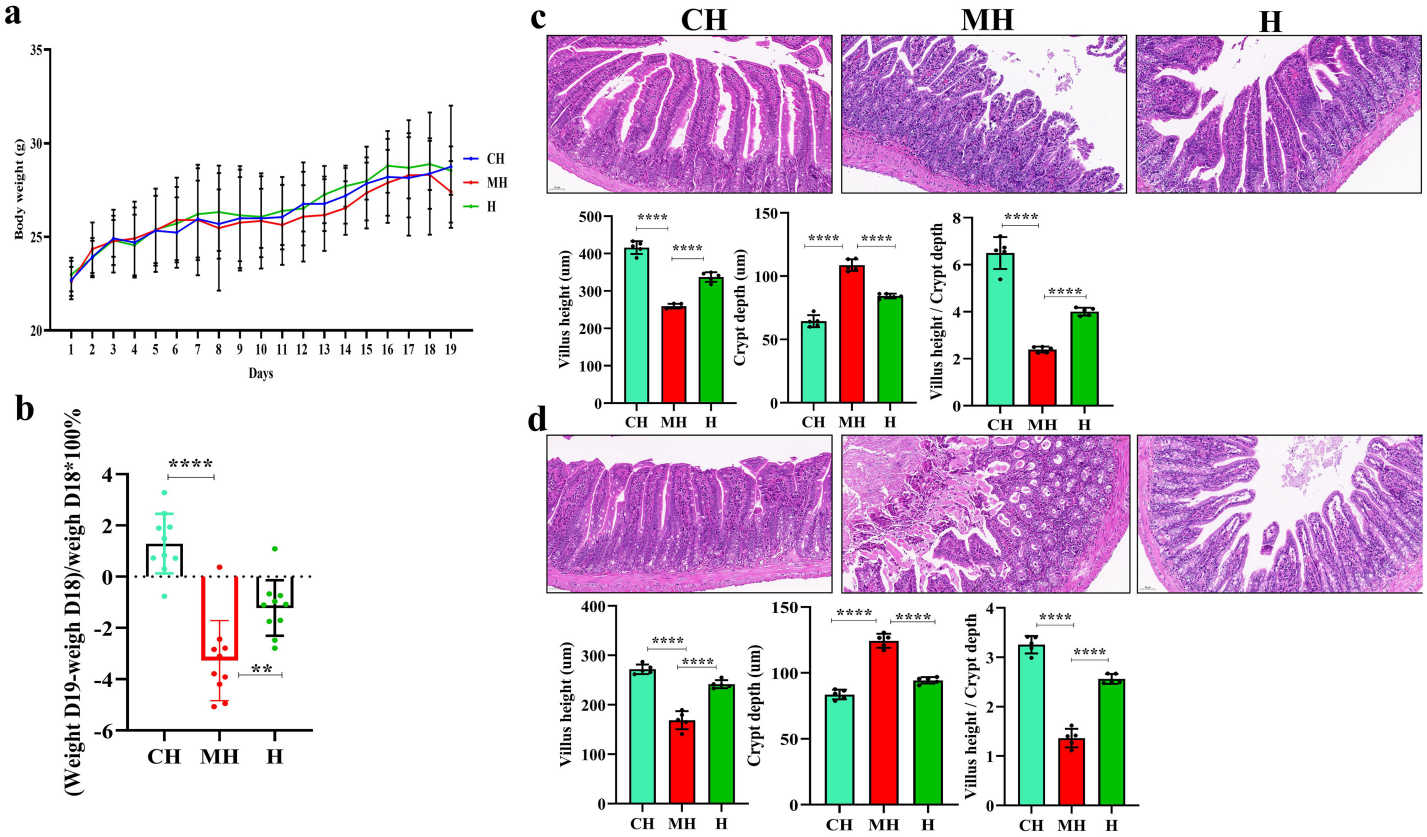
PK remitted intestinal damages in mice induced by LPS. **(A)** body weights, **(B)** weight changes after LPS inducing, **(C)** pathological analysis of the jejunum, **(D)** pathological analysis of the ileum. Scale bar 50 mm. Significance is presented as ^**^P < 0.01 and ^****^P < 0.0001; data are presented as the mean ± SEM.

The body weight of animals in the PK group was marginally higher than that in the CH and MH groups (Figure 1A), and after LPS inducing, the weight of mice in the MH group significantly dropped (*P* < 0.0001), while mice in the PK group had a higher weight (*P* < 0.01) (Figure 1B). Pathological analysis found that the majority of the villi in mice induced by LPS were broken and short, while PK-treated animals had longer and considerably integrated villi. The villi length in the CH (*P* < 0.0001) and H (*P* < 0.0001) groups was longer than that in the M group, and the value of villi length/crypt depth in the MH group was smaller than that in the CH (*P* < 0.0001) and H (*P* < 0.0001) groups, while the crypt depth in the MH was higher than that in the CH (*P* < 0.0001) and H groups (*P* < 0.0001) (Figures 1C, D).

### 3.2 PK-mediated serum oxidation resistance and inflammation levels

**Figure 2:**
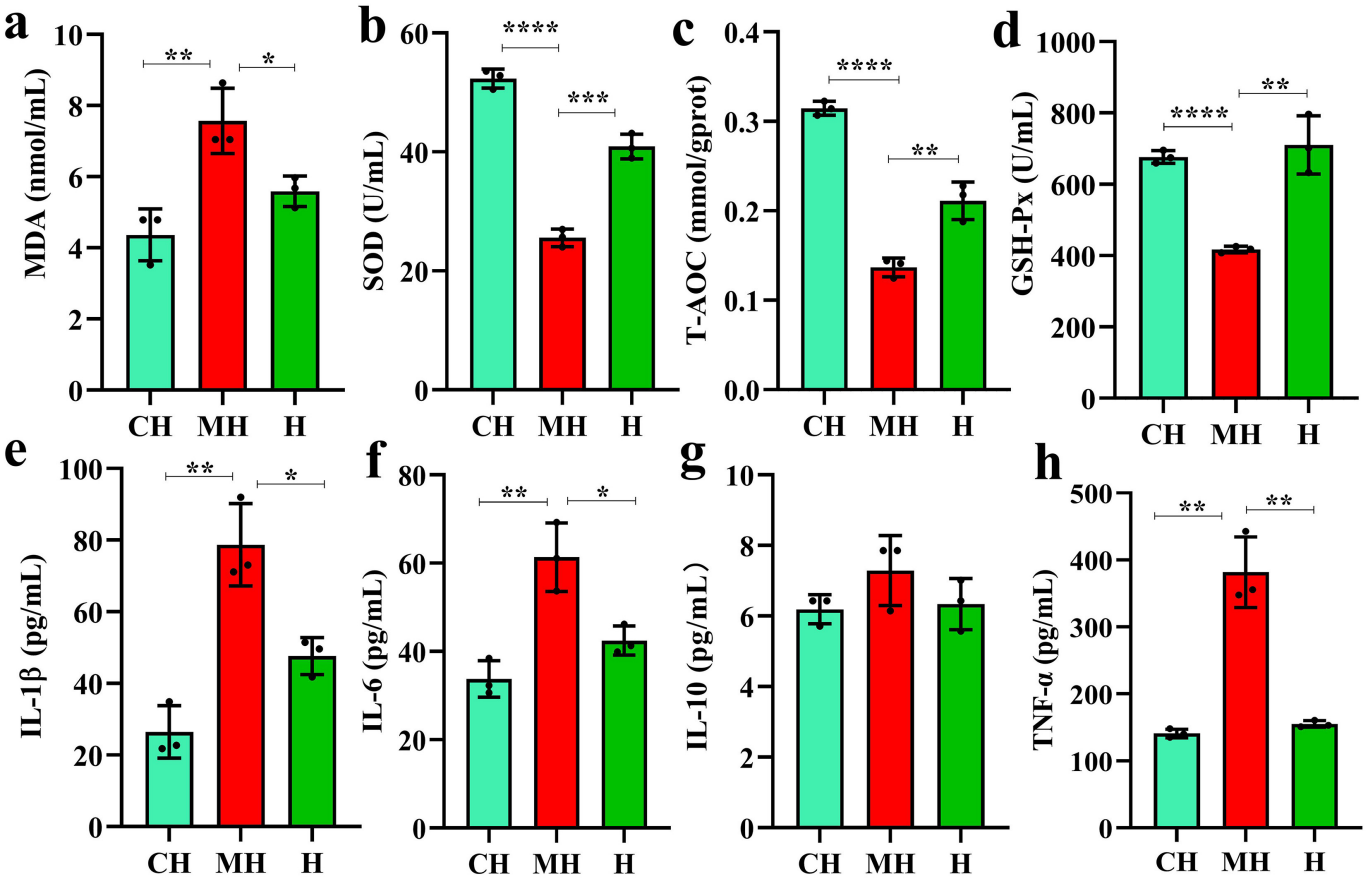
PK-mediated serum oxidation resistance and inflammation levels. **(A)** MDA, **(B)** SOD, **(C)** T-AOC, **(D)** GSH-Px, **(E)** IL-1β, **(F)** IL-6, **(G)** IL-10, **(H)** TNF-α. Significance is presented as *P < 0.05, **P < 0.01, ***P < 0.001, and ****P < 0.0001; data are presented as the mean ± SEM (*n* = 3).

Serum inspection showed that MAD (*P* < 0.05), IL-1β (*P* < 0.05), IL-6 (*P* < 0.05), and TNF-α (*P* < 0.01) were significantly higher in the MH group, while SOD (*P* < 0.001), T-AOC (*P* < 0.01), and GSH-Px (*P* < 0.01) were notably higher in the CH and H groups (Figure 2).

### 3.3 PK partly renovated the composition of intestine microflora in mice

**Figure 3:**
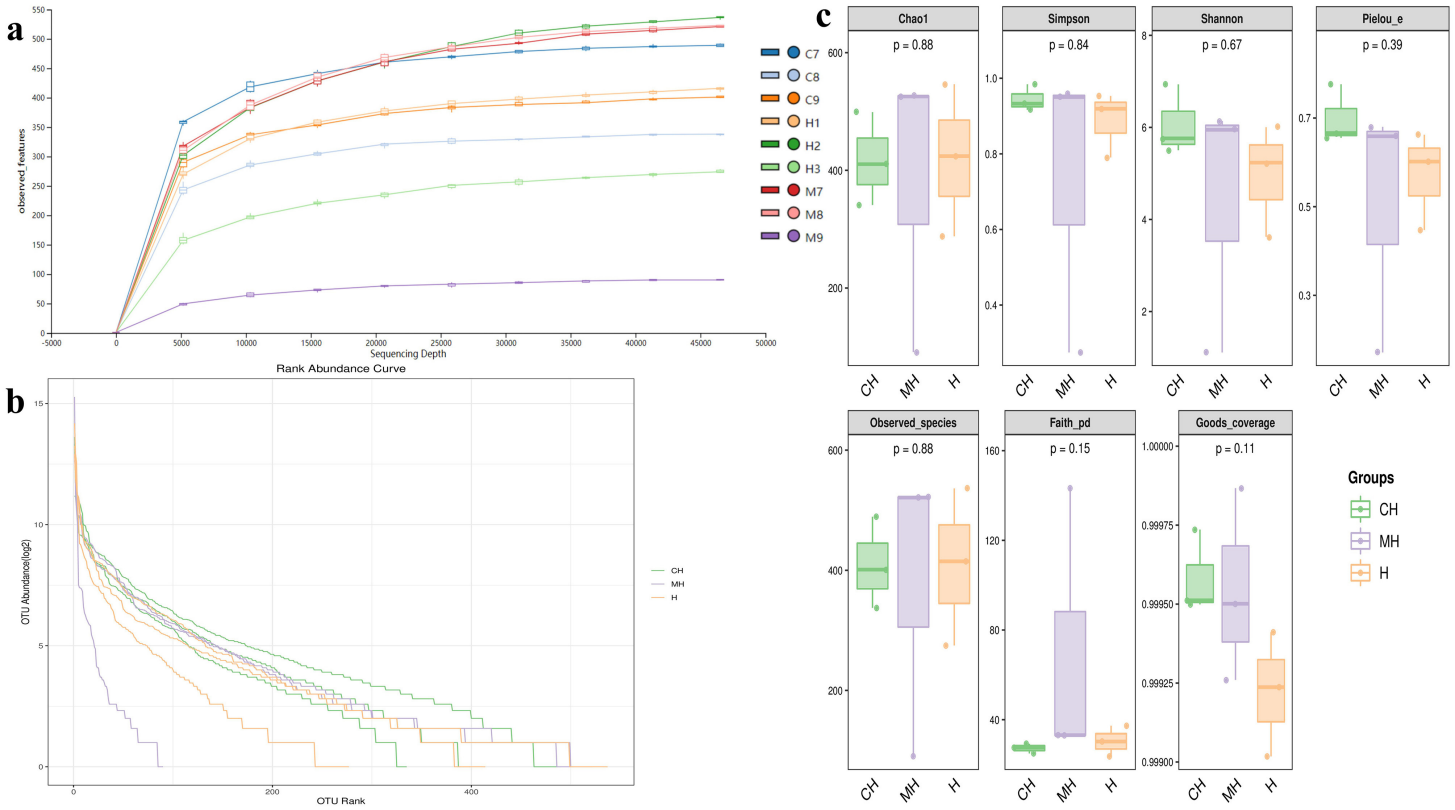
Alpha diversity analysis. **(A)** rarefaction curve, **(B)** rank abundance curve, **(C)** indices.

Over 1CH), 69,600 (MH), and 98,300 (H) raw reads and 66,700 (CH), 57,800 (MH), and 85,000 (H) filtered sequences were identified in mice (Table 1). Alpha diversity calculation found that the rarefaction curves of all mice were horizontal lines after a short rise (Figure 3A), and the rank abundance curves in groups CH, MH, and H were all flat horizontal lines (Figure 3B), which revealed that those mice samples were sufficient with higher evenness. The index result presented no noticeable difference among the CH, MH, and H groups (Figure 3C; Table 2).

**Table 1:** Sequencing data analysis.

**Samples**	**Input**	**Filtered**	**Denoised**	**Merged**	**Non-chimeric**	**Non-singleton**
C7	109,571	94,165	93,457	91,222	89,287	89,276
C8	77,595	66,732	66,220	64,699	62,627	62,623
C9	93,485	80,911	80,129	76,605	69,203	69,191
M7	94,503	81,298	79,642	71,955	54,068	53,984
M8	96,857	82,914	81,468	74,032	49,040	48,964
M9	69,679	57,817	56,896	54,426	54,420	54,413
H1	103,734	89,135	87,890	82,916	70,158	70137
H2	100,679	87,493	86,020	78,166	58,507	58,401
H3	98,374	85,004	84,071	79,914	68,723	68,684

**Table 2:** Alpha diversity index analysis.

**Sample**	**Chao1**	**Simpson**	**Shannon**	**Pielou_e**	**Observed species**	**Faith_pd**
C7	498.923	0.983961	6.93431	0.776175	489.1	29.2839
C8	341.112	0.932804	5.50483	0.655437	337.5	24.949
C9	410.492	0.916575	5.76176	0.666269	401.1	27.543
M7	527.151	0.950349	5.94775	0.659001	521.1	33.0177
M8	525.47	0.958627	6.13969	0.680038	522.2	33.1186
M9	91.2128	0.274758	1.11136	0.17111	90.2	143.277
H1	424.103	0.919352	5.23389	0.601834	414.9	30.2541
H2	546.348	0.953004	6.00414	0.662206	536.3	37.2844
H3	287.919	0.79045	3.62598	0.447504	274.9	23.6465

**Figure 4:**
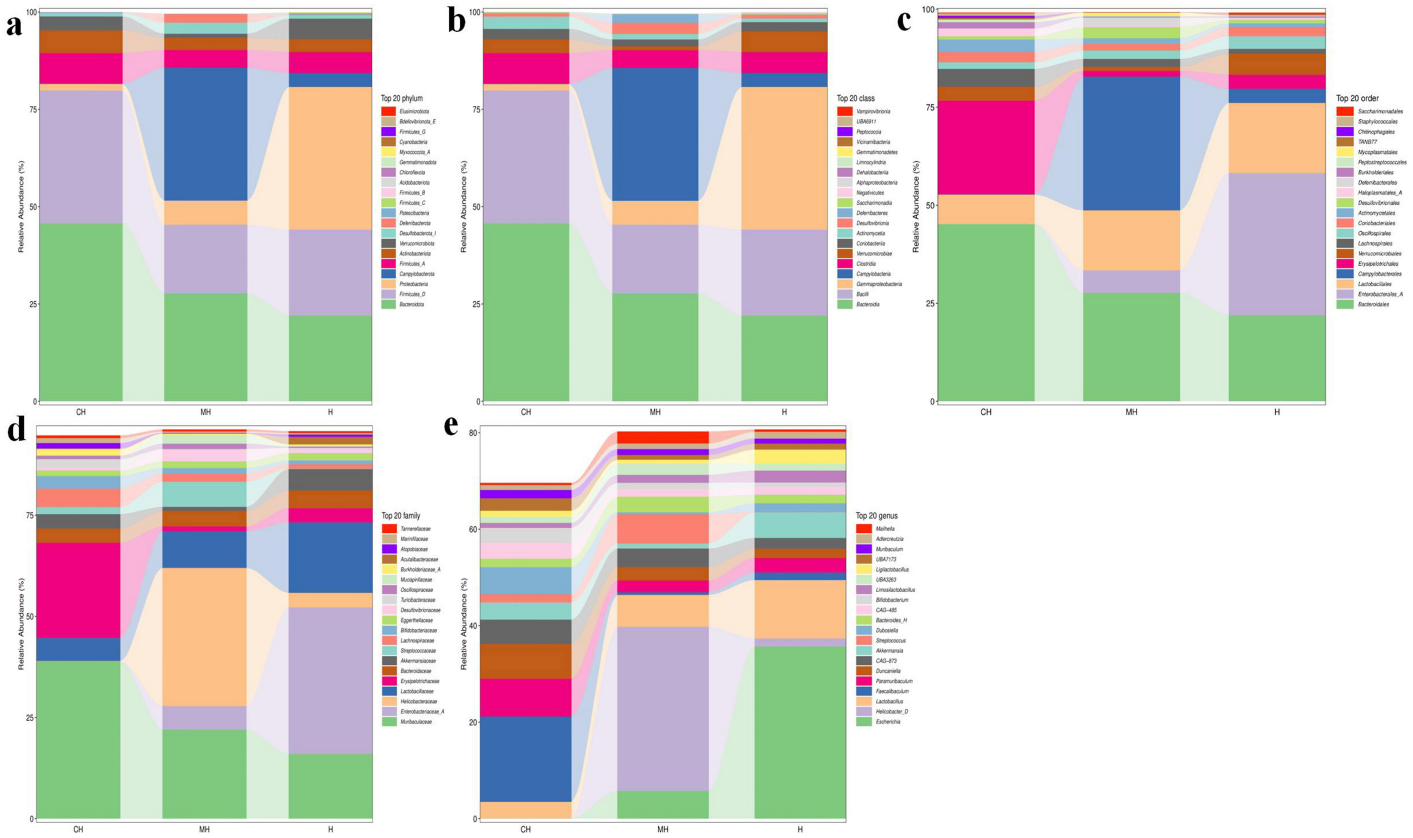
PK-mediated the intestinal microbiota in LPS-induced mice in different taxa. **(A)** phylum, **(B)** class, **(C)** order, **(D)** family, **(E)** genus.

At the phylum level, *Bacteroidota* (45.74*%), Firmicutes*_D (34.09%), and *Firmicutes_*A (7.87%) were the dominating phyla in the CH group, *Campylobacterota* (34.14%), *Bacteroidota* (27.70%), and *Firmicutes_*D (17.67%) were the primary phyla in group MH, while *Proteobacteria* (36.67%), *Firmicutes_*D (22.09%), and *Bacteroidota* (22.00%) were mainly found in group H (Figure 4A). At the class level, *Bacteroidia* (45.74%), *Bacilli* (34.09%), and *Clostridia* (7.87%) were the primary classes in group CH, *Campylobacteria* (34.14%), *Bacteroidia* (27.70%), and *Bacilli* (17.67%) were the primary classes in the MH group, while *Gammaproteobacteria* (36.67%), *Bacilli* (22.09%), and *Bacteroidia* (22.00%) were the primary classes in the H group (Figure 4B). At the order level, *Bacteroidales* (45.16%), *Erysipelotrichales* (23.90%), and *Lactobacillales* (7.49%) were the dominating orders in CH, *Campylobacterales* (34.14%), *Bacteroidales* (27.58%), and *Lactobacillales* (15.30%) were the main orders in MH, while *Enterobacterales_A* (36.28%), *Bacteroidales* (21.94%), and *Lactobacillales* (17.88%) were mainly detected in H (Figure 4C). At the family level, *Muribaculaceae* (38.84%), *Erysipelotrichaceae* (23.46%), and *Lactobacillaceae* (5.75%) were the primary families in CH, *Helicobacteraceae* (34.14%), *Muribaculaceae* (22.03%), and *Lactobacillaceae* (9.07%) were the primary families in MH, while *Enterobacteriaceae_A* (36.27%), *Lactobacillaceae* (17.46%), and *Muribaculaceae* (15.98%) were the main families in H (Figure 4D). At the genus level, *Faecalibaculum* (17.67%), unclassified_*Muribaculaceae* (9.93%), and *Paramuribaculum* (7.86%) were mainly examined in CH, *Helicobacter*_D (34.02%), unclassified_*Muribaculaceae* (6.89%), and *Lactobacillus* (6.60%) were the stable genera in MH, while *Escherichia* (35.67%), *Lactobacillus* (12.10%), and *Akkermansia* (5.31%) were the primary genera in group H (Figure 4E).

### 3.4 PK affected the bacteria abundance in the intestinal flora of mice induced by LPS

**Figure 5:**
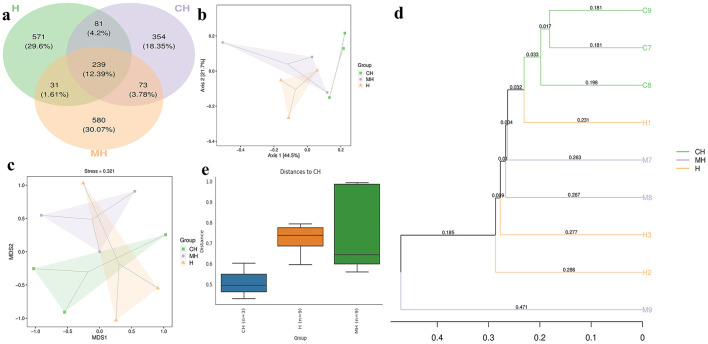
ASV Venn map and beta diversity of the gut flora in mice in different groups. **(A)** Venn chart, **(B)** PCo-A, **(C)** NMDS, **(D)** UPGMA, **(E)** group distance.

**Figure 6:**
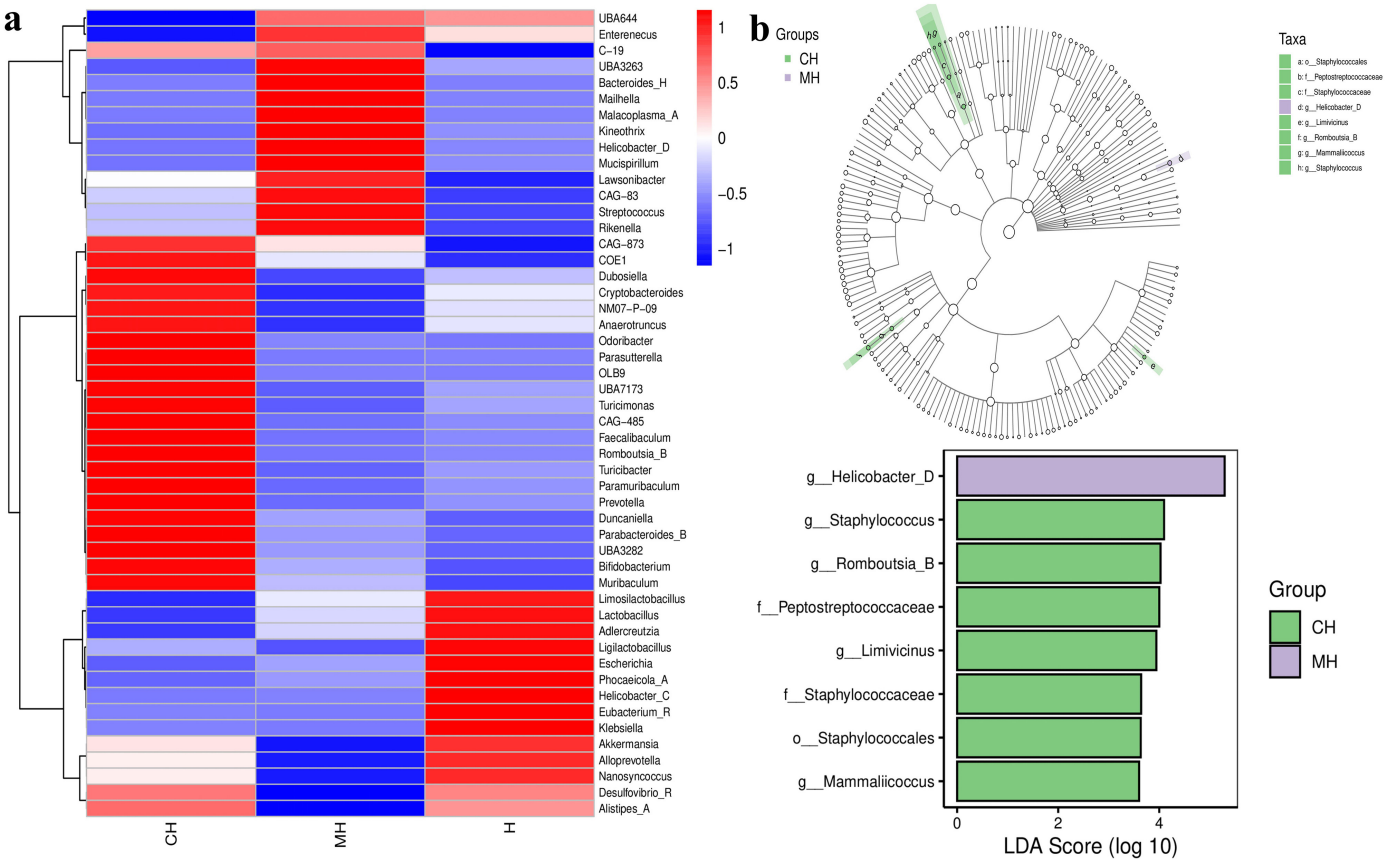
Heatmap and LEfSe analysis of mice microbiota in different groups. **(A)** Heatmap, **(B)** LEfSe.

The Venn graph revealed that there were 2,407 ASVs detected in animals, and 239 ASVs were found in all groups. There were 312 and 328 shared ASVs between CH and MH, and CH and H, respectively (Figure 5A). Beta diversity analysis showed that the distance between CH and H was shorter than between CH and MH; however, it was not significant (*P* > 0.05) (Figures 5B–E). Heatmap analysis showed that *Enterenecus*, UBA3263, *Bacteroides*_H, *Mailhella, Malacoplasma*_A, *Kineothrix, Helicobacter*_D, *Mucispirillum, Lawsonibacter*, CAG-83, *Streptococcus*, and *Rikenella* were higher in MH, *Dubosiella, Cryptobacteroides*, NM07-P-09, *Anaerotruncus, Odoribacter, Parasutterella*, OLB9, UBA7173, *Turicimonas*, CAG-485, *Faecalibaculum, Romboutsia*_B, *Turicibacter, Paramuribaculum, Prevotella, Duncaniella, Parabacteroides*_B, UBA3282, *Bifidobacterium*, and *Muribaculum* were higher in CH, while *Limosilactobacillus, Lactobacilus, Adlercreutzia, Ligilactobacillus, Escherichia, Phocaeucola_A, Helicobacter_C, Eubacterium R*, and *Klebsielia* is changed (Figure 6A). LEfSe showed that *Limivicinus* (*P* < 0.05), *Staphylococcaceae* (*P* < 0.05), *Peptostreptococcaceae* (*P* < 0.05), *Staphylococcales* (*P* < 0.05), *Staphylococcus* (*P* < 0.05), *Mammaliicoccus* (*P* < 0.05), and *Romboutsia*_B (*P* < 0.05) were significantly higher in CH, while *Helicobacter*_D (*P* < 0.05) was significantly higher in MH (Figure 6B).

**Figure 7:**
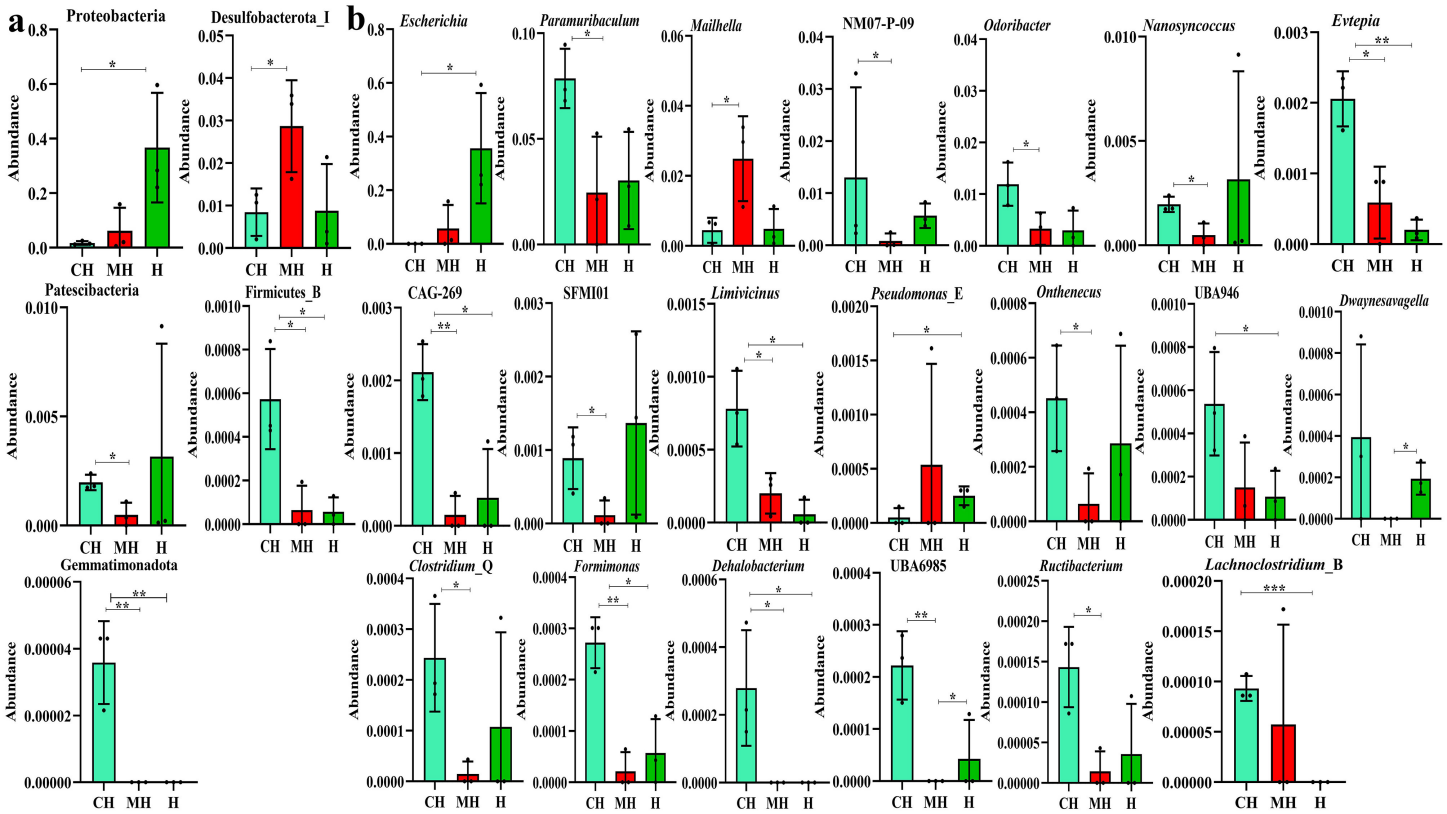
Revealing microbiota differences in mice in different groups using ANOVA analysis. **(A)** phyla, **(B)** genus. Significance is presented as *P < 0.05, **P < 0.01, and ***P < 0.001; data are presented as the mean ± SEM (*n* = 3).

ANOVA analysis showed that compared to group CH, the phylum of Proteobacteria was significantly higher in H (*P* < 0.05), *Desulfobacterota*_I (*P* < 0.05) was markedly higher in group MH, while *Patescibacteria* was significantly higher in group CH than in MH. The enrichment of *Gemmatimonadota* (*P* < 0.01) and *Firmicutes*_B (*P* < 0.05) in animals in groups MH and H was obviously lower than that in CH (Figure 7A). Compared to the genus in group CH, *Escherichia* (*P* < 0.05) and *Pseudomonas*_E (*P* < 0.05) were dramatically higher in H, *Mailhella* (*P* < 0.05) was higher in MH, while *Paramuribaculum* (*P* < 0.05), NM07-P-09 (*P* < 0.05), *Odoribacter* (*P* < 0.05), *Nanosyncoccus* (*P* < 0.05), SFMI01 (*P* < 0.05), *Onthenecus* (*P* < 0.05), *Clostridium*_Q (*P* < 0.05), UBA6985 (*P* < 0.01), and *Ructibacterium* (*P* < 0.05) were significantly lower in MH, and UBA946 (*P* < 0.05) and *Lachnoclostridium*_B (*P* < 0.001) were significantly lower in group H. The abundances of *Evtepia* (*P* < 0.05), *CAG-269* (*P* < 0.05), *Limivicinus* (*P* < 0.05), *Formimonas* (*P* < 0.05), and *Dehalobacterium* (*P* < 0.05) in group CH were significantly higher than those in groups BH and H. *Dwaynesavagella* (*P* < 0.05) and UBA6985 (*P* < 0.05) in group H were significantly higher than those in group MH (Figure 7B).

### 3.5 PK affected the intestinal flora function of mice induced by LPS

**Figure 8:**
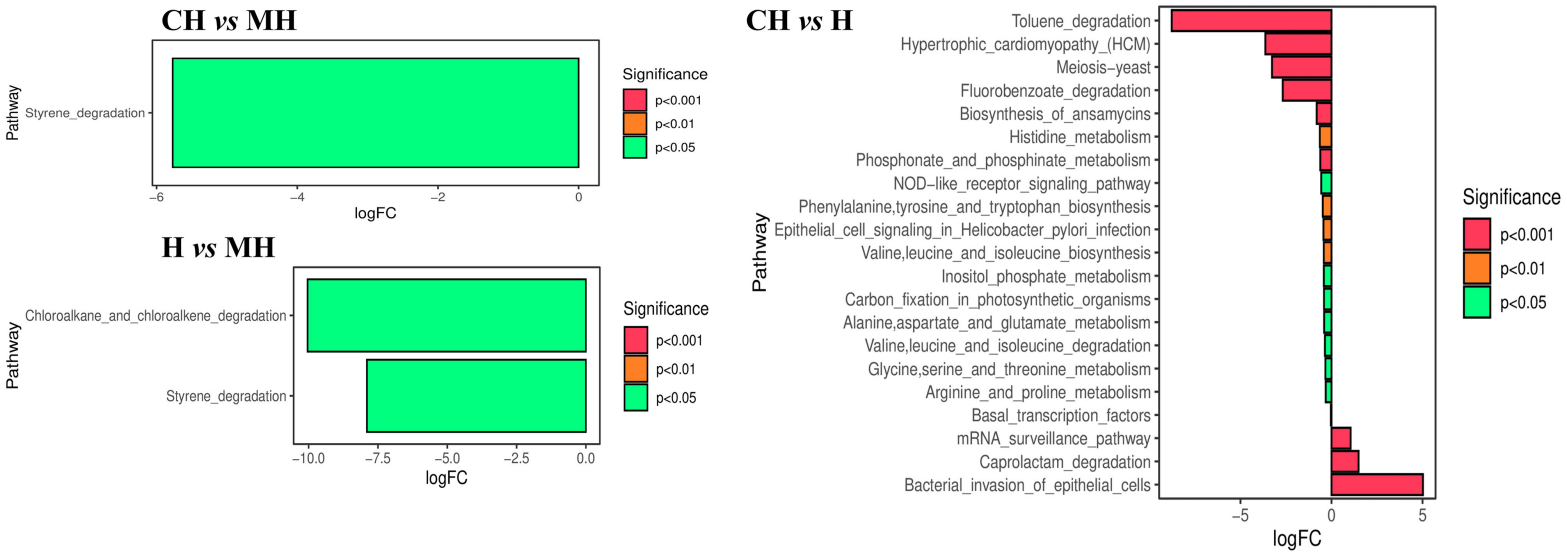
KEGG function analysis of mice microbiota.

The KEGG analysis found that styrene degradation in group CH (*P* < 0.05) and H (*P* < 0.05) was significantly lower than that in group MH. The degradation of chloroalkane and chloroalkene in H was observed to be lower than that in MH (*P* < 0.05). Pathways of bacterial invasion of epithelial cells (*P* < 0.0001), mRNA surveillance pathway (*P* < 0.0001), and caprolactam degradation (*P* < 0.0001) were significantly higher in CH, while toluene degradation (*P* < 0.0001), meiosis yeast (*P* < 0.0001), hypertrophic cardiomyopathy (*P* < 0.0001), for benzoate degradation (*P* < 0.0001), biosynthesis of annamycin's (*P* < 0.001), phosphonate and phosphonate metabolism (*P* < 0.001), histidine metabolism (*P* < 0.01), valine, leucine and isoleucine biosynthesis (*P* < 0.01), epithelial cell signaling in Helicobacter pylori infection (*P* < 0.01), phenylalanine, tyrosine and tryptophan biosynthesis (*P* < 0.01), NOD-like receptor signaling pathway (*P* < 0.05), inositol phosphate metabolism (*P* < 0.05), arginine and proline metabolism(*P* < 0.05), valine, leucine, and isoleucine degradation (*P* < 0.05), basal transcription factors (*P* < 0.05), alanine, aspartate and glutamate metabolism (*P* < 0.05), carbon fixation in photosynthetic organisms (*P* < 0.05) and glycine, serine and threonine metabolism(*P* < 0.05) in H is decreased (Figure 8).

**Figure 9:**
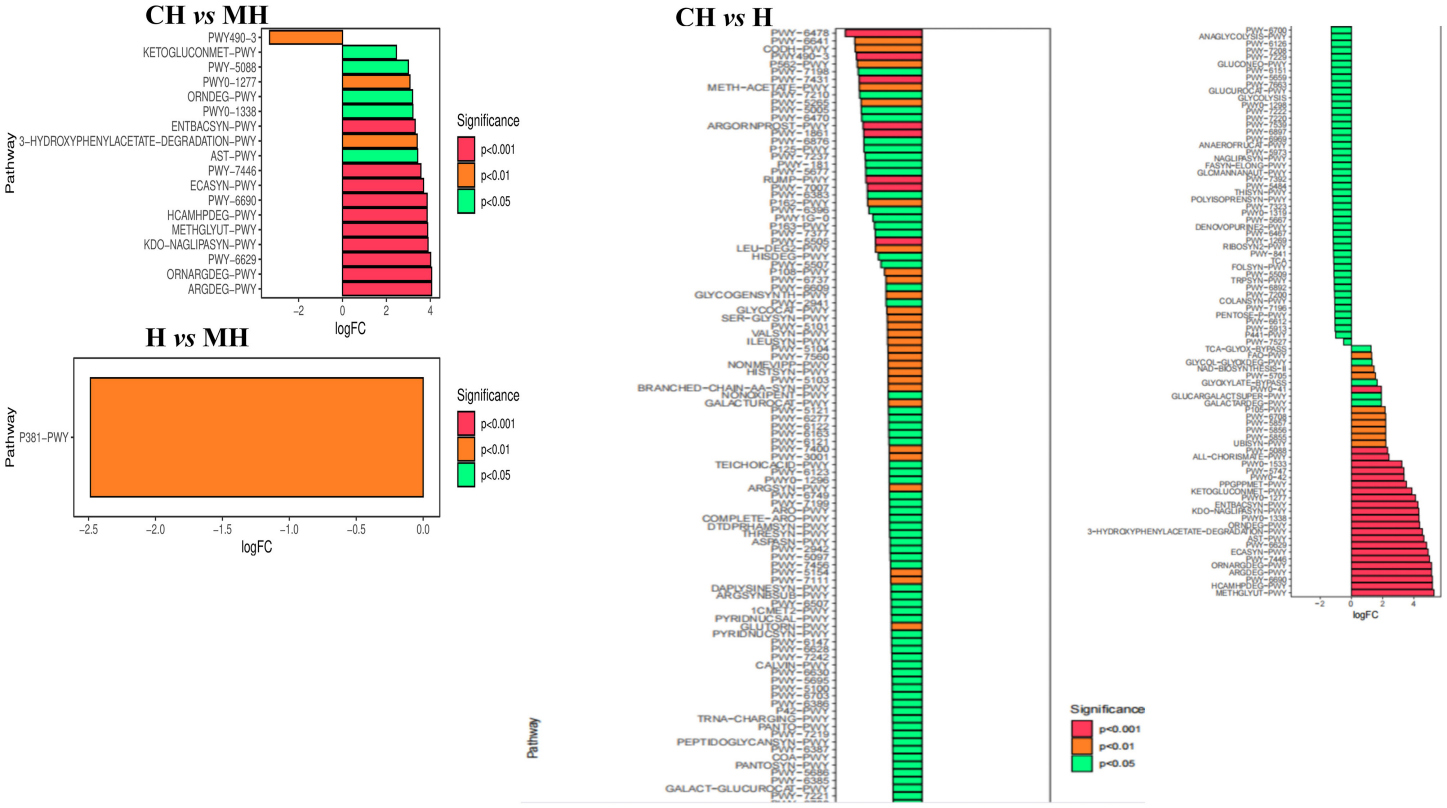
MetaCyc pathway analysis of mice microbiota.

The MetaCyc pathway analysis showed that PWY-6629 (*P* < 0.0001), Argdeg-PWY (*P* < 0.0001), Ornargdeg-PWY (*P* < 0.0001), Methglyyt-PWY (*P* < 0.0001), Kdo-Naglipasyn-PWY (*P* < 0.0001), Ecasyn-PWY (*P* < 0.0001), Entbacsyn-PWY (*P* < 0.0001), Hcamhpdeg-PWY (*P* < 0.001), PWY-6690 (*P* < 0.001), PWY-7446 (*P* < 0.001), 3-Hydroxyphenylacetate-Degradation-PWY (*P* < 0.01), PWY0-1277 (*P* < 0.01), PWY0-1338 (*P* < 0.05), PWY-5088 (*P* < 0.05), Ast-PWY (*P* < 0.05), Ketogluconmet-PWY (*P* < 0.05), and Orndeg-PWY (*P* < 0.05) were markedly higher in group CH, while PWY490-3 (*P* < 0.01) was higher in group MH. P381-PWY in MH was significantly higher in group MH than in group H (*P* < 0.01). There were 186 dramatically different pathways between groups CH and H, such as Methglyut-PWY (*P* < 0.0001), Hcamhpdeg-PWY (*P* < 0.0001), and PWY-6690 (*P* < 0.0001) were significantly higher in group CH, while PWY-7007 (*P* < 0.0001), PWY490-3 (*P* < 0.0001), PWY-6478 (*P* < 0.0001), and others were significantly higher in group H (Figure 9).

## 4 Discussion

Plant polysaccharides are natural macromolecules that have gained increasing attention in pharmacological research (Yin et al., 2019). These polysaccharides exhibit a range of biofunctions, including immune adjustment, antioxidant properties, anti-inflammation effects, anti-tumor activity, and anti-microbe functions (Yin et al., 2019; Wang et al., 2018). Among them, PKP is an important herbal polysaccharide that has received relatively little attention. In this study, we examined the therapeutic effects of PKP on intestinal injuries in mice challenged with LPS, focusing on its antioxidant properties and microbiota composition.

The weigh analysis showed that LPS caused obvious weight loss in mice (Figure 1), which was in agreement with a previous study (Zhang et al., 2019), and PKP could increase the body weight of mice significantly. Pathological results further showed that LPS seriously damaged the villi integrity in mice with shorter villi length and higher crypt depth, while animals supplemented with PKP had better villi with longer villi length and lower crypt depth (Figure 1). Those results demonstrated that PKP could alleviate weight loss in LPS-induced animals by maintaining the integrity of the villi.

Then, we analyzed the serum antioxidant properties and inflammation levels of mice and found that LPS increased the levels of MAD, IL-1β, IL-6, and TNF-α, while it reduced the levels of SOD, T-AOC, and GSH-Px in mice (Figure 2), which were in line with the results reported in LPS-induced animals (Bian et al., 2022; Cao et al., 2018). GSH-Px, SOD, and T-AOC are important antioxidant enzymes inhibiting oxidation damage caused by reactive oxygen species accumulation due to the imbalance of oxidation–reduction homeostasis induced by LPS (Shu et al., 2022). MAD is a well-known biomarker of oxidative stress (Tsikas, 2017). The lower contents of this enzyme and higher levels of antioxidant enzymes in PKP-treated animals showed that PKY could mediate intestinal damage by enhancing antioxidant capacity in mice. IL-1β, IL-6, and TNF-α are commonly recognized pro-inflammatory factors in many pathological reactions (Zheng et al., 2023). The lower levels of those inflammatory factors in PKY-treated mice showed that this polysaccharide could decrease inflammatory response in animals.

Furthermore, we performed microbiome sequencing of mice and obtained 844,477 raw and 725,469 filtered reads (Table 1). There were 2,407 ASVs detected in animals, and there were 312 and 328 shared ASVs between CH and MH, and CH and H, respectively (Figure 5A). A noticeable difference in alpha diversity was not detected in mice, which was in agreement with the results in LPS-stimulated animals (Li et al., 2023a,b), but not in line with the results in LPS-challenged laying hens (Feng et al., 2023) and piglets (Li et al., 2024). In different taxa, LPS changed the structure of microbiota and the potential function of mice, and PKP could partly restore the microbiota composition and function of mice (Figures 4, 8, 9). At the phylum level, the ratio of *Firmicutes/Bacteroidota* was 0.92, 0.64, and 1.00 in CH, MH, and H, respectively. As the *Firmicutes/Bacteroidota* value is accepted as an indicator of dysbiosis (Xu et al., 2023), the current results confirmed that PKP could regulate the microbiota of mice. Finally, we explored the obviously changed bacteria among different mice groups and found 5 phyla and 20 genera of remarkable bacteria (Figure 7). Among them, a higher enrichment of *Mailhella* was examined in *Mycoplasma hyorhinis*-infected pigs (Zhang et al., 2023); the lower enrichment of these genera in group H inferred that PKP could regulate intestinal damage by inhibiting the growth of *Mailhella. A* lower abundance of *Clostridium* was detected in preclinical Alzheimer's disease (Jung et al., 2022). The higher enrichment of these genera in group H showed that PKP could maintain intestine health by promoting the colonization of *Clostridium. Paramuribaculum* is a butyrate-producing and commensal genus (Fang et al., 2023). The higher enrichment of these bacteria in group H revealed that PKP could keep the intestine healthy by promoting the growth of butyrate-producing bacteria.

## 5 Conclusion

We demonstrated that *Polygonatum kingianum* polysaccharide could alleviate intestinal injuries by promoting oxidation resistance, decreasing inflammatory responses, and accommodating the intestinal microbiota of mice. Our results suggest the possibility of developing novel therapies for intestinal diseases.

## Data Availability

The datasets presented in this study can be found in online repositories. The names of the repository/repositories and accession number(s) can be found at: https://www.ncbi.nlm.nih.gov/, PRJNA1149202.
